# Pubertal Suppression for Transgender Youth: A Right to an Open Future Approach in Support of a Youth-Empowered Legal Framework

**DOI:** 10.1007/s11673-025-10493-w

**Published:** 2025-11-20

**Authors:** R. Lee

**Affiliations:** Faculty of Medicine, Imperial College London, London, UK

**Keywords:** Transgender, Puberty suppression, Right to an open future, Gender-affirming care, Trans youth

## Abstract

Irreversible alterations to the form and function of youths’ physical bodies before sexual maturity, and particularly, the potential foreclosure of youths’ fertility options via long-term puberty suppression, is a reason oft-raised in resistance to the provision of puberty blockers (PBs) for minors. Despite the pervasiveness of such assertions by lawmakers and other authorities, the concept of the foreclosure of transgender youths’ future has been subject to surprisingly little philosophical scrutiny in bioethical literature. Joel Feinberg’s “right to an open future” theory provides a foundation for other discussions about childcare decision-making, such as the choice to raise children in a particular religion or to foster their musical talents over their sporting ones. However, relatively fewer attempts have been made to apply Joel Feinberg’s “right to an open future” theory to paediatric transgender medical decision-making. In this article, I consider the relevance of Feinberg’s theory to the context of pubertal suppression for transgender youth and advance in this article reasons why transgender youth should be allowed to make their own decisions about the commencement of puberty suppression to a maximally feasible degree, in order to safeguard their physical health, mental health, autonomy, and capacity for future self-fulfilment.

## Introduction

In recent years, increasing numbers of jurisdictions have adopted laws that ban or severely restrict youth access to pubery blockers (PBs) (Department of Health and Social Care 2024; Simmons-Duffin and Fung 2024). Lawmakers, clinicians, and other fiduciaries often cite the irreversible damage of PBs as justification to ban or severely restrict youth access to gender-affirming care (Brandt v. Rutledge, 551 F. Supp. 3 d 882, 891 [E.D. Ark. 2021], aff’d sub nom. Brandt ex rel. Brandt v. Rutledge 47 F.4th 661 [8th Cir. 2022]). I advance in this article reasons why transgender youth should be allowed to make their own decisions about the commencement of puberty suppression to a maximally feasible degree. To do this, I apply the concept of a “right to an open future.” The concept of a “right to an open future” was introduced by legal and political philosopher Joel Feinberg in 1980 in his seminal work *The Child’s Right to an Open Future* (Feinberg 2015). In it, he describes a set of moral rights that children possess, derived from the autonomy rights that adults possess, that constitute children’s “rights-in-trust,” otherwise known as their “rights to an open future.” This theory has historically been applied to pedagogical and parenting discussions about the best ways to educate a child (Jawoniyi 2014). In addition, a number of bioethicists have applied this concept to paediatric decision-making in, for example, mandatory vaccination (Feldman 2022) and non-therapeutic male circumcision (Darby 2013) for children. This concept has, surprisingly, yet to find much of a toehold in argumentation in support of gender-affirming care for transgender youth. In an article examining the empirical evidence for PBs in their article through the open future lens, Jorgensen et al*.* conclude that treatment pathways that delay decisions about medical transition until adulthood are the most consistent with the open future principle (Jorgensen et al. 2024). This essay provides an alternative view on what is a topical and multifaceted debate.

This article begins with a conceptual delineation of Feinberg’s right to an open future. Following that, I advance the following four arguments in favour of a youth-centred gender-affirming care model: 1) Denying youth the option to commence pubertal blockade has serious and irreversible ramifications. 2) Whatever degree of current autonomy that youth possess should be respected over future rights-in-trust. 3) In cases where youth have limited autonomy, when making a substituted judgement for youth, youths’ preferences should be respected and nurtured to a maximally feasible degree. Finally, 4) I anticipate and respond to a potential objection that the laws around capacity and consent already cover the concerns that the open future model aims to highlight. This essay primarily focuses on examining the rights of transgender youths’ to access PBs at a stage in their childhood when at least some capacity is present.

## Joel Feinberg’s “Right to an Open Future”

Joel Feinberg, in his seminal work *The Child’s Right to an Open Future*, first introduced the concept of a “right to an open future” to legal and philosophical parlance (Feinberg 2015). The conceptual advancement of the right was contextualized within the landmark 1972 U.S. Supreme Court case of *Wisconsin v Yoder* (406 U.S. 205, 207 [1972], “Yoder”)*.* The case involved a First Amendment Free Exercise Clause challenge to a Wisconsin compulsory school attendance law by a group of Old Order Amish. The Amish argued that secular education was antithetical to their religious, agricultural way of life, and could result in the eventual demise of their culture. The Court ultimately ruled in favour of the Amish, after determining that exempting Amish children from the statute was insufficiently harmful to state interests to curtail the religious freedoms of Amish parents. Feinberg disputed the ethical permissibility of the court’s decision. Feinberg found that the Amish were severely constraining their children’s life and career options. Rather, Feinberg was “more sympathetic to the separate concurring opinion in the *Yoder* case, written by Mr Justice White and endorsed by justices Brennan and Stuart, than to the official majority opinion written by the chief justice,” which was that:

A State has a legitimate interest not only in seeking to develop the latent talents of its children but also in seeking to prepare them for the lifestyle that they may later choose, or at least to provide them with an option other than the life they have led in the past. (Feinberg 2015, 150)

The *Yoder* case raises important legal and ethical questions about the scope of parental authority, and the role of the state as *parens patriae.* To what extent are parents at liberty to make parental decisions on the basis of their own values? Should states limit the scope of parental discretion when decisions irrevocably foreclose a child’s future options?

Feinberg explores these philosophical dilemmas in further detail (Feinberg 2015). Feinberg first distinguishes between the moral rights of the adult and the child. Feinberg asserts that a fundamental category of moral rights are held equivalently by both autonomous and non-autonomous individuals, and by both adult and child, termed adult–child rights (*A-C rights*). Autonomous adults possess a set of exclusive rights, known as adult-only rights (*A-rights*), such as the right to consent to sexual intercourse. Child-only rights (*C-rights*) are primarily held by children and comprise two categories. This includes, firstly, dependency rights, or the right to certain goods that are owed on the basis of a child’s dependence on adults for the necessities of life, like the right to sustenance provided by a custodial parent, and secondly, C-rights derived from adult rights to autonomy, known as “rights-in-trust,” or “rights to an open future.” Feinberg describes his conceptualization of “rights-in-trust” in the following manner:

[Rights-in-trust] look like adult autonomy rights, except that the child cannot very well exercise his free choice until later when he is more fully formed and capable. [They are therefore] rights that are to be saved for the child until he is an adult, but which can be violated “in advance,” so to speak, before the child is even in a position to exercise them. His right while he is still a child is to have these future options kept open until he is a fully formed, self-determining adult capable of deciding among them. (Feinberg 2015, 146)

Thus, Feinberg asserts that every autonomy right held by autonomous adults corresponds to a right-in-trust held by children who are presently non-autonomous but who are expected to become so. Such rights-in-trust could be either positive-claims or negative-claims. They could also plausibly contain measures of both elements, such that certain interferences are prohibited and certain actions are prescribed via a singular right.

Scholars, in their interpretation and application of Feinberg’s theory, advance one of two broad conceptions of the right to an open future. The first conception is what philosopher Joseph Millum terms a strong interpretation, in which a child’s right to an open future includes all options that might feasibly be chosen by the autonomous adult that the child could grow into (Millum 2014). Scholars have also advanced a moderate interpretation of the scope of the right. A moderate interpretation requires only that, positively construed, a child is fostered with a reasonable range of skills and opportunities for self-fulfilment as a future autonomous adult, and that, negatively construed, essential options and capacities are not prematurely precluded.

In keeping with the strongly liberal character of Feinberg’s other works, legal scholars find that the open future concept is a manifestation of what Amy Gutmann terms the “liberal argument,” which conceives the concept of the right as prior to the concept of the good. Although this was never explicitly acknowledged by Feinberg, scholars postulate that Feinberg’s open future concept is underpinned by John Rawls’ liberal theory of justice, which conceives of a moral entitlement people have to goods that “have a use whatever a person’s rational plan in life” (Feldman 2022). Feinberg maintains the need for the liberal state to remain neutral to the content of these “goods,” without prioritizing one moral standard over another, say, atheism over theism. The moderate interpretation of the right, in particular, aligns closely with the Rawlsian notion of a set of “goods” to which each person is entitled for self-fulfilment.

## Denying Youth the Opportunity to Commence Pubertal Suppression Creates Greater Foreclosure of Future Options

If a strong interpretation of a child’s rights-in-trust is taken, a recommendation to permit youth to commence PBs should be contingent on a convincing defence that pubertal blockade, when compared to other possible courses of action, maximizes a child’s future capacities and options. Transgender youth, between initial pubertal onset to the start of adulthood, come to identify as a gender other than the one which they were assigned at birth. When youth are confronted by persistent bodily and gender incongruence, and, oftentimes, concomitant feelings of deep distress, they may desire to initiate medical transition in adolescence, alongside a possible social transition. Then, two potential initial courses of action arise: to either commence pubertal blockade or delay it until adulthood. The foreclosure of adolescents’ future options is often alluded to, whether via explicit reference to Feinberg’s rights-in-trust theory or not, in legal and bioethical justifications for parental and state restrictions on youth gender-affirming care. Critics cite the long-term, irreversible loss of adult bone density and fertility options among other potential, under-studied harms of PBs (Jorgensen et al. 2024). It goes without doubt that some transgender youth may experience these unintended side-effects. What Feinberg’s theory necessitates, however, is a comparison of the extent and harms of foreclosure that each option provokes. Realistically, even when fiduciaries attempt to maximize youths’ future options, each choice made for, or by, an adolescent creates its own path dependency, which makes certain futures more probable and others less so. This requires making either a normative or empirical judgement about which course of action preserves maximal rights-in-trust. The following paragraphs juxtapose the extent of foreclosure engendered by each option: 1) the foreclosure of future options via the commencement of PBs is minimal and can be minimized, whereas 2) the foreclosure of future options via the refusal of PBs to transgender youth is significant and irreversible.

### Bone Health

The problem of low bone density, while not ideal, needs to be put into perspective. Firstly, determinants of bone health are multifactorial. Studies that suggest that youths bone density is low during PB treatment also tend to advise caution in definitively or solely attributing such results to pubertal blockade (Klink et al. 2015; Navabi et al. 2021; Lee et al. 2020; Lee 2023). Rather, transgender youth are vulnerable to many crucial risk factors for poor bone health, including a lack of physical activity (Bishop et al. 2020; Espinoza et al. 2022), gender minority stress (Lee 2023; McQuillan et al. 2020; Johns et al. 2021), a lack of exposure to sunlight, and an unhealthy diet (Bishop et al. 2020; Johns et al. 2021). Transgender youth face various barriers to physical activity and outdoor sun exposure, including feeling less safe while playing sport, being prevented from wearing gender non-conforming clothing, and experiencing transphobic language and bullying (Austin et al. 2024). This makes the need for robust biopsychosocial support critical, including support for youth to optimize dietary calcium, take vitamin D supplements, increase weight-bearing exercise, and participate in inclusive physical activity programmes (Lee et al. 2020). In addition, bone health can evolve over time. Regular health assessments over the course of pubertal blockade to evaluate potential contributors to low baseline bone density, such as decreased physical activity and vitamin D status, especially for youth with low baseline bone mineral density, can aid clinicians in identifying bone health issues early and making the necessary recommendations (Lee 2023). Should clinicians find that adolescents with low baseline bone mineral density repeatedly refuse to make lifestyle changes, or that their bone health falls below acceptable levels for a prolonged time period, further discussions can then be had within a supportive carer-practitioner-youth triad about further options.

Additionally, it should be noted that the issue of bone density is rectified to some or full degree via the commencement of oestrogen or testosterone therapy, otherwise known as gender-affirming hormone therapy (GAHT) (Vlot et al. 2017; Stoffers et al. 2019; Schagen et al. 2020; Boogers et al. 2023), the contiguous intervention recommended by the World Professional Association for Transgender Health (WPATH) following puberty suppression (World Professional Association of Transgender Health 2024). The WPATH Standards of Care Version 8 (SOC 8) is a set of international clinical guidelines on the best practices for gender-affirming care. In recent years, there have been critiques about the lack of methodological rigour of the WPATH SOC 8 (Taylor et al 2024), although others have argued, firstly from an empirical viewpoint, that the SOC 8 considers key publications on the topic that may be left undiscussed by other reviews (Grijseels 2024) and that, from a normative viewpoint, the informed care model favoured by the SOC 8 bolsters patient autonomy and beneficence (Surendran et al. 2025). In a study of transgender youth with some of the longest term and most detailed bone measure data, in the group of early- to mid-pubertal transgendered youth who went on to receive thirty-six months of GAHT, mean aBMD and BMAD Z-scores reassuringly increased to higher than baseline (Lee 2023; Schagen et al. 2020). Likewise, in another study of transgender girls, dosages of approximately 4 mg oestrogen achieved adequate serum concentrations to sustain bone mineral density (BMD) (Boogers et al. 2023). In addition, low bone density appears to create a negligible impact on patients’ risk of sustaining fractures when compared to control groups with bone densities in the normal range (Pang et al. 2020).

### Future Fertility

Puberty blockers, medically known as gonadotropin-releasing hormone agonists (GnRH), reduce the secretion of follicle-stimulating hormone (FSH) and leutenizing hormone (LH) in the body, thereby “pausing” sexual maturation in its user. This treatment has long been regarded to have reversible effects on youth fertility and is the standard of care for transgender youth (Coleman et al. 2022; Hembree et al. 2017). The evidence base for the reversibility of GAHT on fertility is weaker (de Nie et al. 2021), which has prompted recommendations by the WPATH for clinicians to discuss fertility preservation options with transgender youth (Surendran et al. 2025), acknowledging the potential for youth regret, the importance of reflecting on fertility goals, and the value of exploring diverse ways to build a family. In clinical practice, fertility counselling often occurs prior to the commencement of PBs rather than GAHT. However, it must be emphasized that the recommendation to discuss fertility preservation options prior to the commencement of pubertal blockade stems from the anticipation that youth may wish to progress to GAHT without the need to pause treatment in order for endogenous puberty to recommence and fertility preservation to take place (Hembree et al. 2017). Even if youth decide not to proceed with fertility preservation prior to pubertal blockade, multiple opportunities for consideration arise during the period of pubertal blockade, and yet again when a decision needs to be made about progression to GAHT—this is likely to be at a point when the youth is much older, with greater decision-making capacity and armed with lived experience of pubertal blockade.

For transgender youth who wish to retain their ability to conceive, a multitude of viable fertility-supporting alternatives are available. Cryopreservation of oocytes or sperm for perimenarchal or postmenarchal transgender youth, or ovarian tissue or testicular tissue cryopreservation for prepubertal youth are options available (Stolk et al. 2023; Rosenthal 2021) and can and have been used for children as young as two years of age undergoing oncological treatment (Cutas and Hens 2014; Rochman 2025; Quinn et al. 2012). Ethical concerns relating to fiduciaries making fertility preservation decisions entirely on behalf of a child, especially one of such a young age, understandably begets further moral quandaries (Cutas and Hens 2014; Klipstein et al. 2020). In this regard, the PB issue is arguably made less contentious by the fact that transgender youth can meaningfully engage in fertility counselling, and fully express their opinions and preferences. Although certain fertility-preserving techniques are still experimental (Slonim et al. 2023), other techniques are established and have been used successfully to treat younger adolescents undergoing chemotherapy and other oncological treatments (Stolk et al. 2023; Cutas and Hens 2014; Rochman 2025). In addition, uterine preservation provides transgender men the option to gestate if desired (Baram et al. 2019). Gestation has been reported in adult transgender men and many opt for the support of surrogate mothers (Obedin-Maliver and Makadon 2015). While many transgender individuals are interested in building a family (Defreyne et al. 2020), evidence suggests that transgender individuals are open towards non-traditional ways to found a family, including adoption, and only a minority desire their own biological children (Stolk et al. 2023). Be that as it may, I agree with Jorgensen et al. that the complexities of the issue of fertility (Jorgensen et al. 2024; Vrouenraets et al. 2022) and financial costs of fertility preservation cannot be understated (Mayhew and Gomez-Lobo 2020; Nahata et al. 2017). Youths, their parents, and their clinical team must consider the delicate trade-off between the potential risks and financial implications of fertility preservation, which are definitely not to be ignored, and benefits of fertility preservation for youth who may wish to conceive of their own biological children.

### Are Transgender Youth Permanently “Locked Into” a Singular Clinical Pathway?

Arguably one of the most significant legal cases on the transgender youth debate both within the United Kingdom and internationally is the judicial review claim in *Quincy Bell and Mrs A v The Tavistock and Portman NHS Foundation Trust and Ors* (*Bell*), which challenged the lawfulness of the consent practices applied by the Gender Identity Development Service (GIDS) to transgender minors seeking access to PBs. In *Bell,* a key theme underpinning the Court’s eventual decision were “matters which are those which objectively ought to be given weight *in the future*.” In particular, the Divisional Court in *Bell* took issue with “the fact that *the vast majorit*y of patients taking PBs go on to GAHT and therefore that s/he is on a pathway to much greater medical interventions” (R (on the application of) Quincy Bell and A v Tavistock and Portman NHS Trust and others [2020] EWHC 3274 (Admin) (Bell (DC)) at [79]–[82]). Jorgensen et al*.,* in an article that raises concerns about the foreclosure of youths’ futures from the administration of PBs, label this phenomenon a “cascade,” whereby an initiating factor is followed by a series of events that “proceed with increasing momentum to a seemingly inevitable conclusion” (Jorgensen et al. 2024).

Proponents of this claim believe that, because a “vast majority” of youth who take PBs proceed to GAHT and surgery, by authorizing youths’ requests for PBs, clinicians set youth on a singular clinical pathway towards lifelong hormone consumption and invasive procedures. The field of “desistance” research examines the prevalence of “persistence” and detransition among transgender youth. Although multiple studies have attempted to shed light on the precise rates of detransition among transgender youth, empirical findings across studies are inconsistent. Moreover, many studies on the topic do not explicitly define “desistance,” or use inconsistent definitions of the term, leaving uncertainty over the accurate rates of adolescent detransition. Even if there is a high rate of “cascading” to GAHT, it offers neither material substantiation that commencing PBs pressures or biases youth towards GAHT, nor insight into youths’ evolving motivations for proceeding with transition. The causal relationship between the commencement of PBs and the eventual commencement of GAHT is distorted by an extraneous, confounding variable, which has formidable influence over both cause and effect—that youth are, authentically and fundamentally, *transgender* and receive benefit from the bodily changes that PBs, and then GAHT, bring to their lives*.* Those who are concerned that pubertal blockade is the sole, main, or even partial motivation for progression to GAHT must also concede that a confounder as weighty as this could easily result in the forging of a spurious correlation where none exists. Those who received PBs, and then GAHT, must have identified as transgender over an extended period of time, especially because age-based restrictions on GAHT are common across jurisdictions, and GAHT is often administered years after initial pubertal blockade. Would an equally, if not more plausible explanation for this “locking in” phenomenon be that transgender youth made discrete decisions across time to advance their embodiment goals? Widely-subscribed healthcare frameworks such as the “clinical pathway” model and the “continuity of care” model guide healthcare professionals in adapting healthcare plans to the evolving needs of the individual patient. These frameworks emphasize the need for a multidisciplinary care plan with time-frame or criteria-based progression through a series of interventions that has been standardized for a specific clinical problem or population. Many health issues require multiple interventions and regular recalibration of plans, and patients generally expect continuation of care in a way that adequately addresses their symptoms. Likewise, the move from PBs to GAHT necessitates clinical consultations on the effects of the treatment on youths’ physical and mental health, and discussions within a multidisciplinary clinical context. It seems, therefore, questionable that transgender youth need to consider the open future implications of *both* pubertal blockade and GAHT prior to the start of PBs, when the two are distinct treatment decisions.

Rather, what appears clear from qualitative research is that detransition can happen across a range of ages and stages of transition, including at the social, medical, or surgical transition phase. Transgender individuals possess enough personal agency to retract a prior decision to initiate transition, even when it may mean facing prospects of new challenges like the relapse of gender dysphoria or the loss of community (Sansfaçon et al. 2023). They evaluate the benefits and risks of each option and eventually decide to detransition if it aligns with their evolving identities, priorities, and needs (Dolotina and Daniolos 2023). Youth who detransition are a heterogeneous cohort, and, therefore, drivers of detransition are manifold (Turban et al. 2021). Detransition is often regarded as synonymous with regret or with a mistaken transition, but the psychosocial realities for youth are often more nuanced. While mourning the loss of their transitioned and pretransition selves, some youth assert continuing to feel “transness” while no longer identifying with the label, express satisfaction with their transition, or emerge with feelings of self-acceptance and personal growth (Sansfaçon et al. 2023). Paradoxically, those who subscribe to a “locked in” interpretation of transgender youths’ transition journeys tend to simultaneously raise concerns over the high rates of “desistance” documented in transition research. Yet, current qualitative findings indicate that transgender individuals, if they come to find transition incongruent with their selfhood, possess the discernment to embark on alternative, nonlinear journeys towards gender actualization, rather than being on a slippery slope towards an inevitable outcome.

Some researchers have pointed out that transgender youth, prior to commencing pubertal blockade, seem already to be of a singular mind in wanting to commence GAHT and surgical transition. Setting aside questions about the empirical accuracy of this claim, youths’ unwavering wish to transition is a potentially meaningful point of discussion in the wider discourse around transgender youths’ access to PBs. However, within the context of an open future analysis, this has neither a direct positive nor negative bearing on their open futures. If youth have “made up their minds” about a so-called “*good*” thing for their open future, their steadfast attitude presumably increases the likelihood that they follow through on the positive undertaking, thus creating a more open future for their adult selves. For example, if an adolescent feels motivated by their favourite fitness and lifestyle “social media influencers” to adhere resolutely to a healthy, balanced diet, or be adamant about not dropping out of school, then parents would likely endeavour to support their decision. Likewise, if youth have made the decision to commence pubertal blockade, and if that decision keeps their future open and promotes self-determination, then there should be no substantive reason to refuse their claim on open future grounds.

### The Foreclosure of Transgender Youths’ Futures When Access to PBs is Denied

On the other hand, empirical evidence demonstrates problematic, and perhaps even more distressing, outcomes for transgender youth who are denied the right to care, arising from the irreversible internal and outward changes associated with endogenous puberty. The World Professional Association of Transgender Health recommends that transgender youth commence PBs during Tanner Stage II of puberty, which begins around the ages of 10 to 11 (2024). When absolute age cut-offs (eg. eighteen or twenty-one) are used by states or by families as proxies for decisional autonomy, minors are precluded from commencing PBs at the appropriate stage, which would have long concluded, or at the minimum, be verging on closure in the vast majority of cases. When transgender youth are forced to undergo puberty of their natal sex, they develop unwanted secondary sexual physical characteristics such as breast tissue growth, internal bone (e.g. hip and jaw) structural changes, vocal changes, and muscle development. In adulthood, many transgender youth who were denied gender-affirming care as minors go on to seek risky, invasive, financially draining and sometimes entirely impossible surgeries in attempts to reverse pubertal changes (Priest 2019; Baker and Restar 2022). Also, in order to access critical care, transgender and gender diverse youth may seek to self-procure medication through unsanctioned sellers (Davis and Marsh 2024; Mosalski 2025). In the absence of medical supervision and proper treatment, this can lead to dosage errors and the consumption of counterfeit medication (Priest 2019). Regrettably, transgender youth who do not receive PBs are at greater risk of poorer mental health, poorer suicide-related outcomes, and self-harm (Turban et al. 2020; Allen et al. 2019; Christensen et al. 2023). Suicide is a tragic and absolute foreclosure of youths’ futures, the risk of which must be taken seriously by lawmakers, fiduciaries, and all who claim to have regard for transgender youths’ well-being and futures. Paradoxically, laws that uncompromisingly foreclose the option of commencing pubertal blockade pre- or peri-pubertally, in an attempt at safeguarding youths’ best interests, place transgender people in a vulnerable position and represent a mishandling of transgender youths’ rights-in-trust.

Granted, the nature of a forward-looking type of right is that whether the future harm we expect may occur to transgender youth will always be unknown in the present. However, rights-in-trust are not violated *only if or when* future harm occurs. In the case of transgender youth, we surely do not need to wait until youth succumb to the physical and mental health ramifications associated with the effects of endogenous puberty of their natal sex to consider their open future rights violated, or at the very least, infringed upon. If fiduciaries make decisions that are likely to, based on the current evidence, foreclose youths’ futures, then the violation of youths’ rights does not occur only when tragedy occurs—it happens right *now*.

## When Rights-in-Trust Take a Backseat: Youth With Capacity Should Have Their Autonomy Respected

In cases where youth capacity is evident, current autonomy should be unequivocally respected over future rights-in-trust. Feinberg (2015) asserts the implausibility of a sharp division between the stages of childhood and adulthood:

Any “mere child” beyond the stage of infancy is only a child in some respects, and already an adult in others. Such dividing lines as the eighteenth or twenty-first birthday are simply approximations (plausible guesses) for the point where all the natural rights-in-trust have become actual [adult] rights. (156)

He also highlights that in a world with a greater understanding of the neurobiological, social, and emotional biomarkers of paediatric neurodevelopment than ever before, it is only natural that the child plays an “ever-greater role in the creation of [her] own life, until at the arbitrarily fixed point of full maturity or adulthood, [she] is at last fully and properly in charge of [her]self” (157). Just as adults with diminished capacity should be, if age were the sole biomarker, autonomous enough to hold A-rights, but in deserving additional safeguarding, instead hold C-rights, there seems to be no principled reason to rule out that some youth 1) possess autonomy commensurate with that of adults or 2) are not fully autonomous but partially autonomous. In the case of 1), the rights of such youth need not be held “in-trust” or “ready-in-waiting” for when the adolescent becomes sufficiently formed and capable. These adolescents are *already* sufficiently mature and should be allowed to make decisions comparable to that of adults. In the case of 2), this nascent autonomy deserves respect, which may entail allowing them to make some decisions for themselves. Developmental and neuroscientific research suggests that the vast majority of youth have at least some degree of capability to engage in complex decision-making independently and have even greater capacities to do so with moral and clinical support from parents and other fiduciaries. They are largely able to apply the “four capacities”—communicating a choice, understanding, reasoning, and appreciation—in decision-making across a wide range of clinically and ethically contentious issues, such as predictive genetic testing, HIV treatment, treatment during hospitalization for acute psychiatric disorders, and the commencement of PBs during adolescence (Marino et al. 2024; Vrouenraets et al. 2021; Clark and Virani 2021). Youth describe their deliberations about commencing PBs as a thoughtful process of weighing pros and cons and by exercising the choice to discuss the decision with trusted people. Many youth and their parents challenge the idea that an adolescent would impulsively chase medical transition and feel frustrated that healthcare professionals do not recognize youths’ capacity for informed decision-making (Crosse 2023; Horton 2022). If many transgender youths have the capacity to consent to PBs, this leads to the natural conclusion that youths’ future autonomy rights cannot be the sole biomarker upon which the commencement of PBs is contingent—alongside youths’ rights to an open future, their *current* autonomy rights should be appropriately evaluated and safeguarded.

## Applying a Substituted Judgement Framework

When considering the decision-making rights of youth with limited current autonomy, the concept of anticipatory autonomy, or substituted judgement, has been proposed. Norvin Richards, for example, suggests that the orientation of an “open future” should be rooted “in terms of what [the adolescent] will be like upon first emerging from their care and beginning life as an adult” (Richards 2018, 98). In making “best guesses” on behalf of youth, the role of parent, clinician, and judge should be, as far as possible, to 1) respect and 2) nurture youths’ decision-making process, rather than to regard the adolescent as merely an object of paternalistic concern.

### Respecting Youths’ Decision-Making Capabilities

On 1), transgender medicine goes to the heart of a patient’s identity, which is an intimate and personal matter. As expounded by bioethicist Florence Ashley in their work on the principle of subsidiarity, decisional authority should only be held by those of higher authorities if lower level decision-makers are incapable of satisfactorily making an informed choice even with strong support and the higher level decision-maker is better positioned to satisfactorily address the issue than lower level decision-makers (Ashley 2022b). In the case of transgender medicine, parents, the courts, and other fiduciaries rarely have as accurate or personal an understanding of youths’ gender struggles or subjectivity as the youths themselves. Due to parents’ and judges’ lack of intimate understanding of the patient’s gender subjectivity, a useful heuristic is to consider whether the reasons against gender-affirming care are so grave that no rational person would choose it regardless of their gender subjectivity (Ashley 2022b). If all transgender youths were making an obviously and objectively poor choice in deciding to initiate pubertal blockade, then intervention by higher-level decision-makers is warranted. The decision to commence puberty suppression is primarily a subjective decision, a *personal* choice that many transgender adults themselves had readily chosen, or would have desired to choose, in their youth (Giovanardi et al. 2019). Granted, inaccurate recollections and negative memory bias should be taken into account, especially if an extended period of time has lapsed since childhood. Nonetheless, according to the principle of subsidiarity as argued by Ashley, the best way to safeguard youths’ future rights-in-trust, in a way that suits youths’ future adult selves best, is to ask youth themselves about their values, cares, and commitments.

The application of this concept of the right to an open future has been analogized to the conferral of legal decision-making rights to surrogates of adult patients in persistent vegetative states. Some raise the objection to the application of this concept to minors by arguing that in the case of the adult with impaired capacity, surrogates can ground their decision in the preferences and opinions of the patient before she lapses into a vegetative state (Dwyer 1994). For minors, a period of prior competence does not exist. Such an argument applies minimally, if at all, to transgender medicine for youth. The epistemological difficulties of understanding minors’ preferences and values become less severe as they enter adolescence. Unlike certain surgical procedures for congenital malformations, for example, that are best performed in early childhood, WPATH recommends that minors commence PBs at Tanner Stage II of puberty, which coincides with early-to-mid adolescence (World Professional Association of Transgender Health 2024). It would be ludicrous to liken a patient in a vegetative state, unable to speak or respond purposefully to external stimuli, with a transgender youth, who is able to fully express their opinions and preferences. Making a substituted judgement about transgender adolescents’ preferences in adulthood can rely on their opinions towards the matter, and should, according to various theories of moral consistency.

### Nurturing Youths’ Decision-Making Capabilities

On 2), rights-in-trust encompasses not just the adolescent’s right not to have their options constrained prematurely but also their capacities, skills, and preferences. Mianna Lotz, for instance, captures here the role that parents play in their child’s personal development: “[Parents have] a duty to provide for their child’s agent-internal conditions by seeking to develop in their child the skills and capacities for information seeking, critical reflection, deliberative independence, and the like” (Millum 2014, 5). The model of the child as a passive creature at the mercy of socialization and biological maturation is emphasized by Feinberg and the model’s advocates. There is no doubt that children have their particular vulnerabilities, for example, to peer influence in their teenage years. However, we cannot turn a blind eye to the realities of developing as an adolescent—that youth can and do shape their own future (Herring 2023). Transgender people face a series of ever and increasingly complex medical decisions as they progress through their lives. This makes the early development of strong capacities for decision-making especially critical and best done with positive support from families, clinicians, and the state. In fostering decision-making skills in lower-level decision-makers with limited capacity, parents and clinicians should encourage them to participate to the greatest extent in conversations about their bodies and potential transition. An adolescent’s natural preferences should also be fostered and attuned to from a young age, in order for them to explore and understand their preferences in-depth and contribute maximally to the adult they become. Youth should be allowed the time, space, and guidance to explore their preferences about their identity and transition options. For example, should youth request one-to-one consultations with an endocrinologist to discuss potential paths forward, or guidance from a genderqueer-affirming therapist, parents should endeavour to support these plans. A greater understanding of youths’ preferences can, then, inform any further decisions about the commencement of gender-affirming care for the adolescent.

## Safeguarding Transgender Youths’ Right to Self-Fulfilment

Considering a moderate interpretation of Feinberg’s right to an open future, otherwise known as the *vital quality* view, the scope of rights-in-trusts narrows to include only certain vital options of moral importance as they pertain to self-fulfilment, characterized by Feinberg as being synonymous to personal well-being. The moderate interpretation is also described by bioethicist Dena Davis as “virtually all the *important rights* we believe adults have, but which must be protected now to be exercised later” (Davis 1997, 9).

In this section, I expand on the self-fulfilment (personal well-being) aspect of Feinberg’s rights-in-trust principle. Here, I apply the “informed desire” theory of well-being to advance the argument that pubertal blockade in adolescence improves youths’ future personal well-being. This concept espouses the fulfilling of one’s freely-formed and informed desires, and the embracing of reasonably pluralistic wants and outcomes (Crisp 2001). Two requirements must be fulfilled for this theory to apply to this context. Firstly, transgender youth need to receive a reasonably complete set of non-evaluative facts about their medical interventions of interest in order to provide informed consent. They should have their decision-making capacities respected and should feel supported within the carer-practitioner-youth triad. Much has been said in this article about the value of nurturing and respecting youths’ capacity from a young age and offering time and guidance to youth who seek support from their caregivers and clinical team.

Additionally, the intervention should allow youth the opportunity to achieve their desires. At first glance, this appears to be an ambitious goal. What, exactly, do transgender youth aim to achieve with pubertal blockade and, more broadly, gender-affirming care? Gender-affirming care has been analogised to abortion and birth control, which are considered *definitional* forms of medical care. Definitional healthcare is “pursued [fundamentally] as a means of defining or actualising fundamental aspects of personal identity” (Ashley 2022a, 133). Those seeking definitional medical care do so primarily because they seek the direct effect of the intervention, rather than to improve their mental or physical health, although this may occur as a by-product of the intervention. In the case of abortion and birth control, the two interventions are widely-used because they successfully induce the termination and prevention of unwanted pregnancies, despite known health risks such as pre-eclampsia and breast cancer, and despite the disruption of the “natural processes” of the body (Ashley 2022a, b). Likewise, gender-affirming care is a form of definitional medical care. The gender-affirming journey is, at its heart, a journey towards empowerment and living life aligned with one’s truest, most authentic self. This journey is often undertaken amidst immense political, social, and familial stigmatization. Ultimately, transgender youth who express the desire to commence pubertal blockade tend to have direct and consistent goals for gender-affirmation; they wish to put a “pause” on endogenous puberty (Davis 1997), thereby placing their “transgender and cisgender hormonal futures in approximate symmetry” (Ashley 2019, 229). In other words, they seek gender-affirmative care because they desire the delay of endogenous puberty and its appearance-related effects.

The achievement of these goals has been shown to yield numerous experiential benefits for transgender youth. Research has demonstrated positive effects of PBs on youths’ self-esteem, school attendance (Horton 2022), body image (Hobson et al. 2024), quality of relationships (Goulding et al. 2023), overall well-being (Jentoft 2019), and the provision of a sense of great relief (Jentoft 2019) from the effects of endogenous puberty. These benefits that youth derive from PBs tend to be specific, individualized, sensory, and harder to quantify (Wright et al. 2025). Therefore, they tend to be less prominent in bioethical discourse compared to more measurable outcomes. Moreover, reviews and commentaries on the evidence base for the benefits of PBs tend to overlook multiple studies that provide evidence for the benefits of gender-affirming care (Noone et al. 2025). In addition, the high degree of consistency in the empirical evidence between studies tends to be disregarded (Horton 2024a). Furthermore, some reviews and commentaries take issue with the lack of randomized controlled trials on the effects of PBs for youth, because randomised controlled trials are considered the gold standard on metrics for the quality of research. However, the need for randomized controlled trials have been refuted elsewhere on the basis that they are methodologically inappropriate for the purposes of the evaluation of gender-affirming care for transgender youth, due to issues with masking, crossover, response bias, and generalizability, among other faults (Ashley et al. 2024).

## Open Future, Competence, and Capacity to Consent

Those who are sympathetic to autonomy-based arguments in favour of transgender youths’ right to pubertal blockade may find that discussions about capacity and competence are well-established and already cover the concerns that the right-to-an-open future highlights. After all, if the application of capacity and open future arguments favour an identical conclusion—that youth deserve access to PBs—might the open future argument be superfluous? I suggest viewing the relationship between capacity and an open future not as overlapping but as *interdependent and mutually supportive.*

Lord Fraser and Lord Scarman’s guidance on the assessment of competence in the seminal *Gillick* case (Gillick V. West Norfolk and Wisbech area health authority. AC. [1986], 112)—widely used in clinical and legal settings—requires not just a mere evaluation of youths’ capacity but also a broader consideration of the youth’s best interests and well-being. The *Gillick* model does not detail the weight that a competent adolescent’s views and their interests should be accorded (Bart et al. 2023). Rather, the framework emphasizes the need for consideration of both and leaves further judgements entirely up to clinical and judicial oversight, which allows some flexibility in the relative weighting of the two concepts based on the individual circumstances of each case. Especially with regard to matters as polarizing as the trans issue, fiduciaries may prefer to give significant weight to a best interests assessment over the competent adolescent’s view. When youths’ broader interests are considered alongside their capacity, the need to appraise the relative merit of various competing interests, and to then decide the direction in which these interests guide the verdict, will form a crucial part of a holistic *Gillick* assessment.

Jeremy R. Garrett, in their work on the open future theory, contends that there is a need to construe the open future principle not as a *right* but as an *interest.* Garrett finds that the invocation of rights language is “exceedingly strong, rigid,” and places the open future principle above all other interests relevant to the child, which does not necessarily result in an optimal outcome for the child (Garrett 2022). Rather, there is a need for a balanced evaluation of various relevant, and sometimes competing, interests. I agree that there is the need to simultaneously consider youths’ various interests alongside the open future concept. Within the medico-legal context, I suggest that an incorporation of the open future framework into the *best interests* decision-making framework for transgender youth will provide a welcome degree of structure with which clinicians and judges can capitalize to make a holistic judgement of youths’ interests. The best interests framework is well-established, ubiquitous in legal and medical settings, and is arguably the most widely-used interests-based framework in paediatric decision-making. The incorporation of the open future model into the best interests framework will necessitate a comparison of the open future principle to other competing interests core to paediatric decision-making, for example, a positive familial relationship and the right to bodily integrity (Herring and Wall 2017), and a juxtaposition of youths’ *current* and *future* interests. There is much that can be said on the weight the open future concept should be accorded when balanced against other competing rights of the child, which is an important piece of conceptual work for future inquiry. However, what I seek to emphasize in this article is that youths’ open futures should be a *foremost* element in any consideration of transgender youths’ interests. As with any intervention with implications for youths’ futures as weighty and complex as this, a careful consideration of youths’ future options is crucial to a fully realized discussion on PBs. In any case, its long-term effects are already a striking point of contention in today’s academic, political, and public discourse, and will likely remain front and centre in discussions about transgender youths’ access to healthcare. In judicial and clinical settings, when judges and clinicians endeavour to make an interests-based assessment for a competent adolescent, taking the findings from this article into account, an alignment of open future and *Gillick* findings promotes certainty about reaching a gender-affirming conclusion and enhances the robustness of the defence of competent youths’ access to PBs.

Correspondingly, there may be cases where youths’ capacity to consent is likely, but marginal doubt or disagreement remains, with the potential that these disagreements escalate to the courts. These disagreements between fiduciaries are conceivable, for example, between members of the youths’ clinical ethics consultation team or between parents and clinicians. In cases like this where marginal doubt remains, a strong case that access to PBs safeguard youths’ right to an open future can provide assurance that PBs are likely to be beneficial to youths’ futures, and provide clarity to cases that rest in the balance.

## Conclusion

This paper applies Feinberg’s right to an open future theory to advance a youth-empowered, gender-affirming model for adolescents who wish to commence puberty suppression. Laws that ban or severely restrict youth access to PBs are ethically unsustainable. They antithetically foreclose potential pathways to current and future trans well-being, often in ways that leave youth from unsupportive families or disadvantaged socioeconomic backgrounds particularly vulnerable. Parents, clinicians, and the state should respect whatever current autonomy that transgender youth possess and endeavour to nurture their decision-making skills and preferences in the cultivation of ever-more bright and open futures.

## Data Availability

Data availability is not applicable for this study. Any further queries can be directed to the corresponding author.
